# A Predictive, Quantitative Model of Spiking Activity and Stimulus-Secretion Coupling in Oxytocin Neurons

**DOI:** 10.1210/en.2017-03068

**Published:** 2018-01-12

**Authors:** Jorge Maícas-Royo, Gareth Leng, Duncan J MacGregor

**Affiliations:** Centre for Discovery Brain Sciences, University of Edinburgh, Edinburgh, United Kingdom

## Abstract

Oxytocin neurons of the rat hypothalamus project to the posterior pituitary, where they secrete their products into the bloodstream. The pattern and quantity of that release depends on the afferent inputs to the neurons, on their intrinsic membrane properties, and on nonlinear interactions between spiking activity and exocytosis: A given number of spikes will trigger more secretion when they arrive close together. Here we present a quantitative computational model of oxytocin neurons that can replicate the results of a wide variety of published experiments. The spiking model mimics electrophysiological data of oxytocin cells responding to cholecystokinin (CCK), a peptide produced in the gut after food intake. The secretion model matches results from *in vitro* experiments on stimulus-secretion coupling in the posterior pituitary. We mimic the plasma clearance of oxytocin with a two-compartment model, replicating the dynamics observed experimentally after infusion and injection of oxytocin. Combining these models allows us to infer, from measurements of oxytocin in plasma, the spiking activity of the oxytocin neurons that produced that secretion. We have tested these inferences with experimental data on oxytocin secretion and spiking activity in response to intravenous injections of CCK. We show how intrinsic mechanisms of the oxytocin neurons determine this relationship: In particular, we show that the presence of an afterhyperpolarization (AHP) in oxytocin neurons dramatically reduces the variability of their spiking activity and even more markedly reduces the variability of oxytocin secretion. The AHP thus acts as a filter, protecting the final product of oxytocin cells from noisy fluctuations.

## Teaser

We present a model of oxytocin neurons that relates secretion to spike activity. We fit this to experimental data and use it to explore the significance of intrinsic membrane properties.

Magnocellular oxytocin neurons in the supraoptic nucleus (SON) and paraventricular nucleus of the hypothalamus project their axons to the posterior pituitary, where they secrete their hormones into the bloodstream. Oxytocin has an indispensable role in breastfeeding and an important one in parturition (1), but the secretion of oxytocin is also regulated by a variety of metabolic signals arising from the gastrointestinal tract, and in the rat, oxytocin secretion also regulates sodium excretion and gut motility (2).

The membrane properties of these neurons have been studied extensively by electrophysiological studies *in vitro* (3–6). In these neurons, spikes are typically triggered by the arrival of excitatory inputs [excitatory postsynaptic potentials (EPSPs)] from diverse brain areas. Whenever a spike is produced, Ca^2+^ enters the cell through voltage-activated channels and subsequently activates K^+^ channels that mediate postspike hyperpolarizations. Large conductance channels open and close rapidly, producing a short hyperpolarizing afterpotential (HAP), which makes the neurons relatively inexcitable for 30 to 50 ms (7). Small conductance channels produce a medium afterhyperpolarization (AHP). This is much smaller than the HAP, but the half-life is much longer (about 350 ms), so the AHP accumulates over successive spikes, and the resulting level of activity-dependent hyperpolarization will reflect the average level of spike activity over the preceding few seconds (7). Some oxytocin neurons also generate an activity-dependent depolarizing afterpotential (8), but this is usually quite small and masked by the larger activity-dependent hyperpolarizations.

The patterning of spikes generated by these neurons has also been studied extensively *in vivo* (4, 9–11). In lactating rats, suckling induces brief intense bursts of spikes in oxytocin cells, but other stimuli produce graded increases in spike activity. For example, intravenous (i.v.) injections of cholecystokinin (CCK) produce a dose-dependent increase in spike activity that lasts for 10 to 15 minutes (12–16), producing a transient increase in plasma oxytocin. CCK is secreted from the duodenum in response to a meal and acts at CCK1 receptors on gastric vagal afferents; these project to neurons in the nucleus tractus solitarii, which in turn project directly to magnocellular oxytocin neurons (17, 18). The subsequent secretion of oxytocin is thought to regulate gut motility and sodium excretion at the kidneys (19, 20).

The spontaneous spiking activity of oxytocin neurons can be matched by a modified leaky integrate-fire model, which incorporates a HAP and an AHP (7, 21). This model can closely match the statistical features of spike patterning in oxytocin neurons, as reflected by the interspike interval distribution and the index of dispersion of spike rate. Given this, it should be possible to use the model to infer the synaptic input that oxytocin cells receive when responding, for example, to CCK, if we assume that the CCK-evoked input consists solely of a change in excitatory input rate.

Our previous work indicates that the AHP in oxytocin neurons, by acting as an activity-dependent negative feedback, reduces the second-by-second variability (index of dispersion) in the firing rate of oxytocin cells (21). Because of particular features of stimulus-secretion coupling in these neurons, this “regularization” of firing rate is likely to be most important during dynamic challenges to oxytocin cell activity. In oxytocin neurons, secretion is a nonlinear function of spike activity: a given number of spikes secrete more oxytocin when they are close together than when sparsely distributed. This nonlinearity is marked: during a reflex milk ejection, oxytocin cells fire about 100 spikes in just 2 seconds (22), and during this burst, each spike releases, on average, about 100 times as much oxytocin as spikes that occur at the typical basal firing rate of 2 spikes/s (23). Because of this nonlinearity, the oxytocin secretion from a single cell depends not only on its mean firing rate, but also on the variability of its firing rate, due to the disproportionate influence of high firing rate fluctuations.

The mechanisms of stimulus-secretion coupling are complex, but we recently published a model of stimulus-secretion coupling in magnocellular vasopressin cells fitted to data on stimulus-evoked vasopressin secretion (24). The properties of vasopressin terminals differ quantitatively from those of oxytocin terminals, and here we modified the vasopressin secretion model to fit the properties of oxytocin terminals (25–27). Combining the spiking model of oxytocin neurons with this secretion model allows us to model the activity-dependent output of oxytocin cells. To predict the consequences for plasma concentrations, we also introduced a model of the clearance of oxytocin from plasma. For this, there is good historical data (28–30). Applying this allows us to predict, from the model, the plasma oxytocin concentration that will result from a given stimulus to the oxytocin cells. In the case of CCK, again there is published data to compare with the model. This allows us to assess the importance of the AHP not only for spike activity but also for the important biological signal—the resulting change in plasma oxytocin concentration.

## Methods

**Figure 1.**
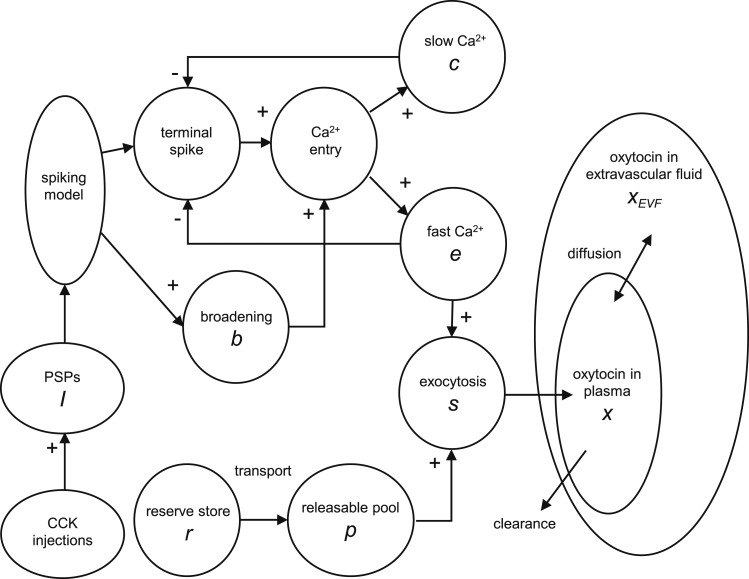
The combined spiking, secretion, and diffusion model. The integrate and fire–based spiking model responds to randomly arriving PSPs. The i.v. injection of CCK is simulated as an exponentially decaying increment in the mean arrival rate of EPSPs. The resulting spikes become the input of the secretion model. In that model, spike-induced Ca^2+^ entry at secretory terminals is positively modulated by activity-dependent spike broadening (*b*), and negatively modulated by fast (*e*) and slow (*c*) Ca^2+^ variables that inhibit spike-induced opening of Ca^2+^ channels (24). The secretion rate (*s*) is the product of the releasable pool (*p*) and *e*. When depleted, pool *p* is refilled from a reserve pool (*r*) at a rate dependent on the pool content. Oxytocin in plasma (*x*) is cleared with an exponential decay and diffuses between the plasma and extravascular fluid (*x_EVF_*) according to the concentration gradient.

We used a previously described model for the spiking activity of oxytocin neurons (24; parameters given in Table 1) and adapted a published model of stimulus-secretion coupling in vasopressin neurons to model oxytocin secretion (24). We also added a two-compartment model to mimic the dynamics of oxytocin concentration in plasma (Fig. 1). The models were developed using software written in C++ and a graphical interface based in the open-source wxWidgets library. Simulations were run for up to 10,000 seconds using a 1-ms step.

**Table 1.** Spiking Model Parameter Values

Name	Description	Value	Units
*I_re_*	Excitatory input rate	292	Hz
*I_ri_*	Inhibitory input rate	292	Hz
*epsp_h_*	EPSP amplitude	2	mV
*ipsp_h_*	IPSP amplitude	−2	mV
*λ_syn_*	PSP half-life	3.5	ms
*k_HAP_*	HAP amplitude per spike	30	mV
*λ_HAP_*	HAP half-life	7.5	ms
*k_AHP_*	AHP amplitude per spike	1	mV
*λ_AHP_*	AHP half-life	350	ms
*V_rest_*	Resting potential	−56	mV
*V_thresh_*	Spike threshold potential	−50	mV
*λ_CCK_*	CCK half-life in plasma	230	s
*CCK_dur_*	CCK injection duration	20	s
*k_CCK_*	CCK i.v. injection	20	µg/kg

**Figure 2.**
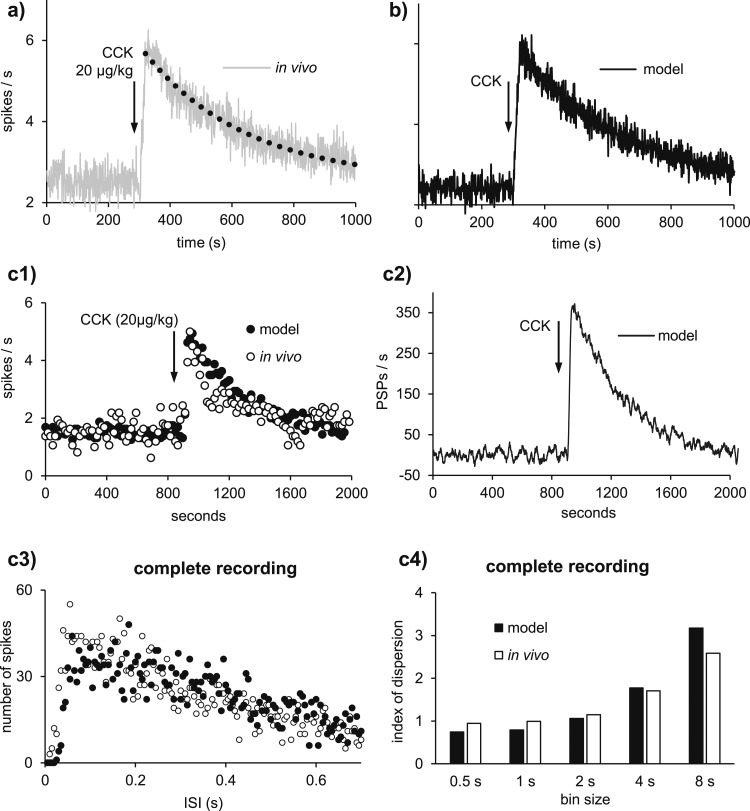
Responses of oxytocin neurons to CCK and simulation in model cells. (a) The average response (in 1-second bins) of 23 oxytocin neurons to i.v. injection of 20 µg/kg CCK. The response decays exponentially with a best-fit half-life of 230 seconds (dotted line). (b) Response of a model oxytocin cell to a simulated challenge with CCK, simulated as an increase in PSP rate that decays exponentially with a half-life of 230 seconds, matching the measured half-life of CCK in plasma. The simulation was run 23 times with different random seeds, and the figure shows the average (see Tables 1 and 2 for model parameters). (c1) A typical response to CCK from a single oxytocin cell (white dots). With a model cell (black dots) that has a HAP and an AHP, it is possible to match this response closely. Parameter values as in Table 1 except *I*_*re*_ = *I*_*ri*_ = 210 Hz. (c2) In this simulation, CCK increases the EPSP rate: The increase decays exponentially with a half-life of 230 ms. (c3) The interspike interval distribution, constructed from the complete activity shown in (c1), shows how often two consecutive spikes have a particular distance between them; the distribution from the model cell (black dots) closely matches that of the recorded cell (white dots). (c4) The index of dispersion measures longer timescale spike patterning, showing how spike rate variability changes using different bin widths, again from the entire activity shown in (c1). The model (black bars) closely matches the values measured in the recorded oxytocin neuron (white bars).

Parameters of the integrate-and-fire based spiking model, with values chosen from Maícas Royo *et al*. (21) and CCK parameters, as used for the simulations in Fig. 2b.

### Spiking model

The integrate and fire–based spiking model (21) simulates the firing activity of oxytocin cells in response to EPSPs and inhibitory postsynaptic potentials (IPSPs). We model postsynaptic potentials (PSPs) as arriving Poisson randomly at mean rates *I_re_* and *I_ri_*, and in this study, we fixed *I_ri_* to be equal to *I_re_*. Thus, the time an EPSP arrives, *epsp_time_* [Eq. (1)], is defined by:

$$epsp_{time}=\frac{-\log\left(1-rand\right)}{I_{re}},\;\;\text{where}\;rand\;\text{is}\;\text{a}\;\text{random}\;\text{number}\;\text{between}\;0\;\text{and}\;1$$ (1)

The IPSP arrival times follow the same formula.

### Responses to CCK

We mimicked the effect of i.v. injection of CCK by adding an additional random EPSP contribution, with mean rate *I_rCCK_*. The total EPSP rate is the sum of *I_re_* and *I_rCCK_*.

The increase in EPSP rate during simulated CCK injection follows a linear function *k_CCK_*/*CCK*_dur_ defined by the quantity of CCK injected, *k_CCK_*, and the duration of the injection, *CCK*_dur_ [Eq. (2)].

We assume that CCK is cleared from plasma following an exponential decay with time constant *τ_CCK_* [Eqs. (2) and (3)]:

dIrCCKdt=(kCCKCCKdur)−IrCCKτCCK  when CCKstart≤t≤CCKstart+CCKdur (2)

$$\frac{dI_{rCCK}}{dt}=\frac{-I_{rCCK}}{\tau_{CCK}}\;\;\text{when}\;t>CCK_{start}+CCK_{dur}$$ (3)

where *CCK_start_* is the injection’s start time.

Time constants [Eq. (4)] are calculated from half-life parameters by:

$$\tau_{x}=\frac{\lambda_{x}}{\ln\left(2\right)}$$ (4)

We fixed the magnitude of EPSPs and IPSPs, *epsp_h_* and *ipsp_h_*, at 2 mV, having an opposite sign for EPSPs and IPSPs. The final input depends on the number of inputs, *epsp_n_* and *ipsp_n_*, per unit of time (fixed at 1 ms in our simulations) [Eq. (5)]. *epsp_n_* is the number of EPSPs obtained in a given time unit from a random process with mean rate *I_re_*.

$$I=epsp_{h}.epsp_{n}+ipsp_{h}.ipsp_{n}$$ (5)

*V_syn_* represents the contribution of synaptic input to the membrane potential *V* and decays to 0 with time constant *τ*_syn_ corresponding to a half-life *λ_syn_* of 3.5 ms [Eq. (6)].

$$\frac{dV_{syn}}{dt}=-\frac{V_{syn}}{\tau_{syn}}+I$$ (6)

Initially, the model neuron is at a resting potential, *V_rest_* = –56 mV. If inputs summate to increase the membrane potential *V* above a threshold *V_th_* = –50 mV, the neuron produces a spike. Then, the model triggers a HAP and an AHP, and *V* evolves according to [Eq. (7)]:

$$V=V_{rest}+V_{syn}-HAP-AHP$$ (7)

*HAP* [Eq. (8)] has a fixed amplitude (*k_HAP_* = 30 mV) and a time constant, *τ_HAP_*, that corresponds to a half-life of 7.5 ms, following values from previous work (21). *AHP* [Eq. (9)] also has a fixed amplitude (*k_AHP_* = 1), and *τ_AHP_* was set to correspond to a half-life *λ_AHP_* of 350 ms as used previously (21); we explored different values of *k_AHP_* in the range of values (0.2 to 1.4) found previously from fits to individual oxytocin neurons (21); the results were qualitatively similar for other values of *k_AHP_*:

$$\frac{dHAP}{dt}=-\frac{HAP}{\tau_{HAP}}+k_{HAP}\cdot \delta$$ (8)

$$\frac{dAHP}{dt}=-\frac{AHP}{\tau_{AHP}}+k_{AHP}\cdot \delta$$ (9)

where δ = 1 if a spike is fired at time *t* and δ = 0 otherwise.

### Secretion model

The secretion model is an adaptation of the model of MacGregor and Leng (24), developed to mimic stimulus-secretion coupling in vasopressin neurons. When spikes invade the secretory terminals, exocytosis occurs at sites close to clusters of voltage-gated Ca^2+^ channels. These sites experience transiently high Ca^2+^ concentrations in response to spikes, but the Ca^2+^ swiftly diffuses into the cytosol (31). This is represented by making secretion proportional to a “fast” Ca^2+^ variable *e*. At increased frequencies, the spikes broaden (32, 33), producing a larger rise in *e*. The resulting facilitation of secretion is limited by activity-dependent attenuation of secretion, modeled as arising from Ca^2+^-dependent inactivation of Ca^2+^ channels in the submembrane compartment, and by activation of Ca^2+^-dependent K^+^ channels. The model consists of differential equations that take as input the spike events generated by the spiking model. Variables representing spike broadening (*b*), cytosolic Ca^2+^ concentration (*c*), and submembrane Ca^2+^ concentration (*e*) are all incremented with each spike. The Ca^2+^ variables model Ca^2+^-mediated signals at specific action sites: We did not attempt to represent the full dynamics of intracellular calcium changes.

We model spike broadening (*b*) [Eq. (10)] by increasing it by *k_b_* = 0.021 when a spike arrives and with an exponential decay with half-life *λ_b_ =* 2 seconds:

$$\frac{db}{dt}=-\frac{b}{\tau_{b}}+k_{b}\cdot \delta$$ (10)

where δ = 1 if a spike is fired at time t and δ = 0 otherwise.

The Ca^2+^ entry, *Ca_ent_*, provoked by spikes has two other effects. *c* and *e* measure how the concentration of Ca^2+^ changes at the cytosol and in the submembrane compartment. They are incremented by *k_c_* = 0.0003 [Eq. (11)] and *k_e_* = 1.5 [Eq. (12)] with every spike and decay with half-lives *λ_c_* = 20 seconds and *λ_e_* = 100 ms:

$$\frac{dc}{dt}=-\frac{c}{\tau_{c}}+k_{c}\cdot Ca_{ent}\cdot \delta$$ (11)

$$\frac{de}{dt}=-\frac{e}{\tau_{e}}+k_{e}\cdot Ca_{ent}\cdot \delta$$ (12)

where δ = 1 if a spike is fired at time *t* and δ= 0 otherwise.

Ca^2+^ entry [Eq. (13)] depends on spike broadening (*b*) and is subject to Ca^2+^-dependent inhibition:

$$Ca_{ent}=e_{inhib}\cdot c_{inhib}\cdot (b+b_{base})$$ (13)

The basal level of spike broadening is given by *b_base_* = 0.5. Ca^2+^ entry is inhibited by *c* [Eq. (14)] and *e* [Eq. (15)] using two inverted Hill equations with threshold and coefficient parameters *c_θ_* = 0.14, *e_θ_* = 12, *c_n_* = 5, and *e_n_* = 5:

$$c_{inhib}=1-\frac{c^{c_{n}}}{c^{c_{n}}+c_{\theta }{}^{c_{n}}}$$ (14)

$$e_{inhib}=1-\frac{e^{e_{n}}}{e^{e_{n}}+e_{\theta }{}^{e_{n}}}$$ (15)

The releasable vesicle pool (*p*) [Eq. (16)] is depleted with secretion, *s*, and refilled when not full (*p_max_* = 5 ng) at a rate proportional to the remaining reserve pool (*r*) divided by its maximum capacity (*r_max_* = 1 µg). The refill rate is scaled by *β* = 120:

$$\frac{dp}{dt}=-s+\beta \cdot \frac{r}{r_{max}}\;\;\;\text{when}\;p<p_{max},\;\;-s\;\text{otherwise}$$ (16)

The reserve pool [Eq. (17)] is depleted exponentially as it refills *p*, with its maximum (initial) value defined by *r*_max_:

$$\frac{dr}{dt}=-\beta \cdot \frac{r}{r_{max}}\;\;\text{ when }p<p_{max},\;\;0\;\text{otherwise}$$ (17)

The rate of secretion (*s*) [Eq. (18)] is the product of *e* raised to the power *ϕ* (because of the cooperativeness of the Ca^2+^ activation of exocytosis) (34), the releasable pool (*p*), and a scaling factor (*α*):

$$s=e^{\phi }\cdot \alpha \cdot p$$ (18)

The parameters of the vasopressin model were fitted to data obtained for secretion from the whole neural lobe, containing the axons of up to 9000 neurons (35, 36). Thus parameters relevant to quantities of secretion (*p*, *p_max_*, *r*, and *r_max_*) from the whole population are about 9000 times higher than would be appropriate for single cells. The same approach was taken here, and the same “correction factor” applies.

To adapt this model to match oxytocin secretion, we made six changes from the parameters of the vasopressin model (24): (1) We decreased *k_b_* from 0.05 to 0.021, reducing the spike broadening. (2) We reduced the sensitivity to Ca^2+^ entry in the submembrane compartment, by increasing *e_θ_* from 2.8 to 12. (3) We increased *c_θ_* from 0.07 to 0.14. (4) We reduced the cooperativeness of the Ca^2+^ activation of exocytosis from *ϕ* = 3 to *ϕ* = 2. (5) We increased *α*, a scaling factor, to 0.003 to match the levels of oxytocin measured in plasma. (6) We increased *β*, the refill rate of the pools from the reserve, from 50 to 120. The new parameters are given in Table 2.

**Table 2.** Secretion Model Parameter Values, Modified From The Parameters Used for a Model of Vasopressin Secretion (24)

Name	Description	Value	Units
*k_b_*	Broadening per spike	0.021	—
*λ_b_*	Broadening half-life	2000	ms
*b_base_*	Basal spike broadening	0.5	—
*k_c_*	Max cytosolic Ca^2+^ per spike	0.0003	—
*λ_c_*	Cytosolic Ca^2+^ half-life	20,000	ms
*k_e_*	Max submembrane Ca^2+^ per spike	1.5	—
*λ_e_*	Submembrane Ca^2+^ half-life	100	ms
*c_θ_*	Threshold, terminal inhibition by *c*	0.14	—
*c_n_*	Gradient, terminal inhibition by *c*	5	—
*e_θ_*	Threshold, terminal inhibition by *e*	12	—
*e_n_*	Gradient, terminal inhibition by *e*	5	—
*β*	Pool refill rate scaling factor	120	pg/s
*r_max_*	Reserve store maximum	1000	ng
*p_max_*	Reserve pool maximum	5	ng
*α*	Secretion scaling factor	0.003	pg/s
*φ*	Cooperativeness of the Ca^2+^ activation of exocytosis	2	—

The results mimic the secretion of the entire population by considering that the average response of the population can be mimicked by the response of a single oxytocin cell multiplied by a scaling factor.

### Two-compartment diffusion model

To simulate how the oxytocin that enters the plasma (*x*) is cleared, we developed a two-compartment model [Eq. (19)]. Secreted oxytocin enters the plasma volume (*C_plasma_*), and is cleared from it mainly through the kidneys and liver. The second compartment represents the extravascular fluid compartment (*C_EVF_*), and oxytocin diffuses between these two compartments according to the concentration gradient. The clearance from plasma and the diffusion between compartments follow exponential differential equations with a clearance half-life, *λ_clr_*, of 68 seconds and a diffusion half-life, *λ_diff_*, of 61 seconds (Table 3), values derived from experimental reference data.

**Table 3.** Diffusion Model Parameter Values

Parameter	Description	Normal Rat	Kidneys or Splanchnic Clamped	Both Clamped	Normal Rat
	Experiments	Fabian *et al.* (29)			Ginsburg and Smith (28)
	Plasma oxytocin half-life (s)	126	380	480	99
	Total fluid volume (mL)	18.25	37.5	35.75	No data
	Duration of oxytocin infusion (s)	1800	—	—	2
*λ_clr_*	Clearance half-life (s)	68	135	188	68
*λ_diff_*	Diffusion half-life (s)	61	—	—	—
*C_plasma_*	Plasma volume (mL)	8.5	—	—	—
*C_EVF_*	Extravascular fluid volume (mL)	9.75	—	—	—
	Plasma oxytocin half-life (s)	126	379	479	91

The top section gives values measured experimentally. The middle section gives parameter values used in the diffusion model. The bottom line shows model measurements from simulations of the experiments.

$$\frac{dx}{dt}=s-\frac{x}{\tau_{clr}}-\;\frac{DiffRate}{\tau_{diff}}$$ (19)

The oxytocin content in plasma (*x*) [Eq. (19)] and extravascular fluid (*x_EVF_*) [Eq. (20)] change due to diffusion between the compartments following the oxytocin concentration gradient (*DiffRate*) [Eq. (21)]:

$$\;\frac{dx_{EVF}}{dt}=\frac{DiffRate}{\tau_{diff}}\;$$ (20)

DiffRate= (xCplasma− xEVFCEVF)⋅Cplasma+CEVF2 (21)

### Reference data

To fit the spiking model, we used a library of recordings of oxytocin neurons in urethane-anesthetized rats. Full details of these experiments have been published previously (15, 16, 37). In brief, neurons were recorded from the SON using a transpharyngeal surgical approach, and were antidromically identified as projecting to the posterior pituitary. Oxytocin neurons were identified by their excitatory responses to i.v. injection of CCK, and spike times were collected using Spike2 software (Cambridge Electronic Design Ltd.). Model data were compared with recorded spike activity by comparing the interspike interval distributions (in 5-ms bins) and by comparing the index of dispersion of firing rate, calculated as the ratio of variance to mean rate for bin widths of 0.5, 1, 2, 4, and 8 seconds.

To fit the secretion model, we used data from three independent data sets: (1) In Bicknell *et al.* (25) and Bicknell (26), oxytocin release from isolated rat posterior pituitaries was measured by radioimmunoassay after: 20-minute periods of 13-Hz stimulation; 18, 36, 54, and 72-second periods at 13 Hz; and 156 pulses at 6.5, 13, 26, and 52 Hz. (2) In Bondy *et al.* (27), rat posterior pituitaries were stimulated with 600 pulses at 1, 4, 8, 12, 20, and 30 Hz. The released oxytocin was measured by radioimmunoassay and normalized to release evoked by 600 pulses at 12 Hz.

To fit the diffusion model, we matched data from experiments in rats described by Fabian *et al.* (29) and Ginsburg and Smith (28), who measured plasma oxytocin by bioassays. Ginsburg and Smith (28) reported that in male rats, a 440 ng/100 g bolus injection of oxytocin disappears from plasma with an apparent half-life of 1.65 ± 0.13 minutes (1.73 ± 0.1 minutes in female rats). Fabian *et al.* (29) found that, after a constant 30-minute infusion of oxytocin at rates between 550 and 13,200 pg/min/100 g body weight, plasma oxytocin concentrations fell to 50% of the initial value in a median time of 126 seconds, and continuously infused oxytocin was distributed in an apparent volume of 7.3 mL/100 g body weight. Assuming a plasma volume of 3.4 mL/100 g body weight, we calculate a plasma compartment *C_plasma_* = 8.5 mL for a 250-g rat and an extravascular fluid compartment *C_EVF_* = 9.75 mL. Fabian *et al.* (29) measured the peak concentrations just before they stopped their infusions (Table 3). Ginsburg and Smith (28) measured the peak value 1 minute after the injection of 440 ng/100 g oxytocin in male rats (average 46 ng/mL). Finally, we mimic the clearance found in rats with the kidneys or the splanchnic area clamped and with both areas clamped (Tables 3 and 4).

**Table 4.** Peak Concentration for the Two-Compartment Diffusion Model

	Oxytocin	Peak Concentrations, ng/mL	
Fabian *et al.* (29) 30-minute infusion			
		Fabian *et al.* (29)	Model
	0.550 ng/100 g/mL	0.220	0.270
	3 ng/100 g/mL	—	1.447
	13.2 ng/100 g/mL	6.160	6.347
Ginsburg and Smith (28) injection			
		Ginsburg and Smith (28)*^a^*	Model
	440 ng/100 g	45.32 (±6.27) ng/mL	43.48

^a^ After 60 s.

To fit the combined model, which includes the spiking, secretion, and diffusion, we used four sets of plasma measurements of oxytocin from independent experiments in which rats were given an i.v. injection of CCK: Conscious virgin female rats, in which blood samples were taken before and after i.v. injection of 20 µg/kg CCK (38). Conscious male rats in which blood samples were taken before and after i.v. injection of 10 µg/kg CCK (19). Two groups of anesthetized female rats in which blood samples were taken before and after i.v. injection of 20 µg/kg CCK (39).

## Results

### Reference data

We selected 23 recordings from oxytocin neurons that showed a fast and clear response to CCK and in which the firing rate subsequently returned to the initial level. The 23 cells had a mean [standard deviation (SD)] spontaneous firing rate of 2.5 (0.39) spikes/s (range, 0.02 to 7.9 spikes/s), measured as the average over 4 minutes before injection. The cells responded to i.v. injections of 20 µg/kg CCK with a mean increase of 1.46 (0.74) spikes/s (range, 0.57 to 3.6 spikes/s), measured as the difference between the basal firing rate and the average over the 5 minutes after the injection (Fig. 2a). The decay of the mean response from 50 seconds after injection was well fitted by a single exponential equation with a half-life of 230 seconds (*R*^2^ = 0.88) (Fig. 2a).

### Spiking model

For the spiking model, we chose a basal mean PSP rate of 292 Hz (basal EPSP rate is equal to basal IPSP rate) to match the spontaneous firing rate of 2.5 spikes/s. After 5 minutes of basal activity, we simulated an injection of CCK as a linear increase in mean EPSP frequency over 20 seconds that declined exponentially to the basal EPSP rate with a half-life of 230 seconds. These values gave a close match to the average response profile of the reference set of oxytocin neurons to CCK (Fig. 2b). Figure 2b shows the average of 23 runs of the spiking model. The variability of this average is less than the variability of the average of the reference data: The 23 model neurons are all identical and firing at the same mean rate, whereas the neurons in the reference data differed in intrinsic properties and mean firing rates.

The spiking model reproduces various statistical characteristics of single oxytocin cells (Fig. 2c). Thus, for the neuron shown in Fig. 2, by modifying only the basal PSP rate (Table 1), the model matches the mean spike rate over the whole recording (Fig. 2c1), simulating the firing rate increment response as an increase in EPSP rate that modifies the oxytocin neuron spiking activity (Fig. 2c2). The model also matches the interspike interval distribution (Fig. 2c3) and mimics the index of dispersion of the firing rate during the complete recording, which measures the variability of the spike rate at different bin widths (Fig. 2c4).

### Secretion model

To model stimulus-secretion coupling, we modified the previously published vasopressin secretion model (24) to match data from three experiments where oxytocin secreted from isolated posterior pituitaries was measured. We mimicked these protocols in the spiking model, progressively adapting the secretion model to fit the oxytocin data by changing six parameter values (*k_b_*, *c_θ_*, *e_θ,_β*, *α*, and *ϕ*).

**Figure 3.**
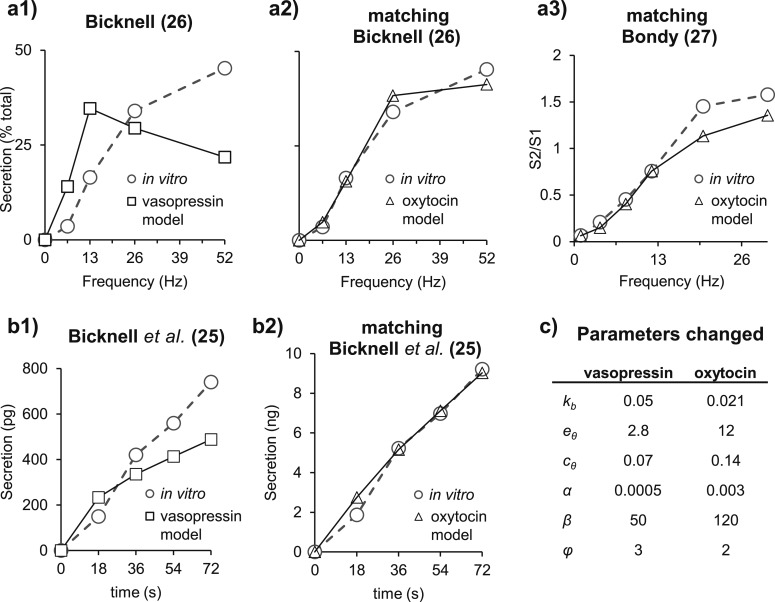
Oxytocin secretion model dynamics. Oxytocin secretion follows a nonlinear function of the spiking activity. We adapted a previous model of vasopressin secretion (white squares) (24), modifying six parameter values to obtain our oxytocin model (white triangles). With those changes, we matched the oxytocin secretion from experiments (white circles). (a1) In Bicknell’s (26) experiments, when posterior pituitaries were electrically stimulated with 156 pulses at 6.5, 13, 26, and 52 Hz, vasopressin secretion (white squares) was maximal at 13 Hz. By contrast, oxytocin secretion (white circles) continued to increase up to 52 Hz. (a2) Matching the data from Bicknell (26) with the oxytocin model. (a3) Using the same parameters, we also obtained a good match to data from Bondy *et al.* (27). In those experiments, glands were stimulated with 600 pulses at 1, 4, 8, 12, 20, and 30 Hz, and evoked secretion (S2) was expressed as a ratio of S1, a reference secretion produced by a preceding stimulus at 12 Hz. (b1) In Bicknell *et al.* (25), glands were stimulated at 13 Hz for 18 to 72 seconds: Vasopressin secretion peaked during the first 18 seconds and showed subsequent fatigue (white squares), whereas oxytocin showed a consistent response (white circles). (b2) We matched that response with the model using the same parameters as in (a2) and (a3). (c) The six parameters changed from the model of vasopressin secretion (24).

In the first of these experiments (26), 156 pulses at 6.5, 13, 26, and 52 Hz were applied to the posterior pituitaries (Fig. 3a1). The second source of experimental data (27) followed a similar protocol, this time stimulating with 600 pulses at 1, 4, 8, 12, 20, and 30 Hz (Fig. 3a3).

In the third set of data (25), isolated rat posterior pituitaries were stimulated *in vitro* at 13 Hz for 18, 36, 54, and 72 seconds in a randomized order (Fig. 3b1). This third set of data is critical for estimating the temporal profile of secretion, and it showed that, unlike vasopressin secretion, which shows fatigue, oxytocin secretion is relatively stable over time in response to a constant frequency of stimulation. The modified model fits all three sets of data well (Fig. 3a2, 3a3, and 3b2).

**Figure 4.**
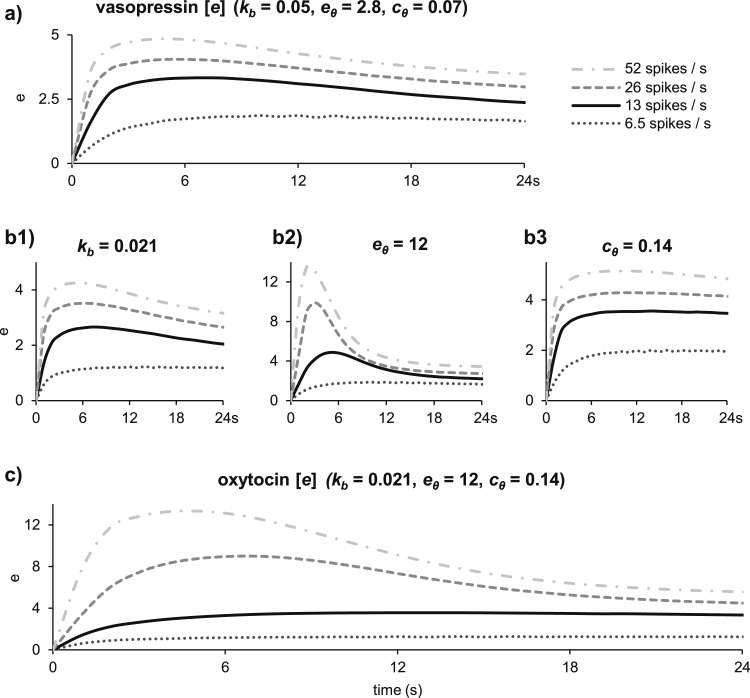
Transition from a vasopressin secretion model to an oxytocin secretion model. In both models, secretion is proportional to the submembrane Ca^2+^ concentration (*e*). We show here how *e* changes during 24 seconds of stimulation at constant rates of 6.5, 13, 26, and 52 spikes/s. (a) In the vasopressin model, for stimulations at 13, 26, and 52 spikes/s, *e* reaches a peak during the first 2 seconds of stimulation, followed by fatigue. Neither the fatigue, nor the early peak, is present at 6.5 spikes/s. To obtain the frequency facilitation seen in Fig. 3, we need to eliminate the fatigue at 13 spikes/s and increase the response at higher frequencies. (b1) Decreasing *k_b_* produces less saturation at high frequencies, increasing the difference between responses. (b2) Increasing *e_θ_* increases the peak response, especially at higher spike rates, but does not reduce the fatigue. (b3) Increasing *c_θ_* eliminates the fatigue but does not separate the responses to different stimulation frequencies. (c) Combining these three changes reproduces the frequency facilitation of secretion while eliminating fatigue at 13 spikes/s.

How the changes in model parameters were arrived at is illustrated in Fig. 4. In the vasopressin model, the submembrane Ca^2+^ concentration (*e*), which has a direct role in exocytosis [Eq. (19)], displays fatigue at a firing rate of 13 spikes/s, and the rate of secretion declines during constant stimulation (Fig. 4a). This is inconsistent with the experimental oxytocin data. In addition, vasopressin secretion per pulse declines at frequencies above 13 Hz, whereas oxytocin secretion is facilitated. Reducing the broadening of spikes, *k_b_*, (Fig. 4b1) reduces secretion at low frequencies and increases secretion at high frequencies by reducing Ca^2+^-induced inhibition of Ca^2+^ entry and reduces but does not eliminate the fatigue. Increasing *e_θ_* to weaken the Ca^2+^-induced inhibition of Ca^2+^ entry enhances secretion, particularly at high frequencies (Fig. 4b2), but fatigue is much more prominent after this change. Raising *c_θ_*, to reduce the sensitivity to cytosolic Ca^2+^, reduces Ca^2+^-induced inhibition of Ca^2+^ entry and so eliminates fatigue. Combining these three changes matched the frequency response (Fig. 4c). To match the slope of the temporal response to a constant frequency of 13 Hz, we also needed to change the exponent in the secretion equation, *ϕ*, from 3 in the vasopressin model to 2, indicating a smaller cooperative activation of exocytosis (34).

The constant factor, *α*, scales the output of the model quantitatively to measured oxytocin levels. In rats, milk ejection bursts typically contain about 100 spikes over about 2 seconds and release about 1 mU (2.2 ng) of oxytocin (22). Setting *α* = 0.003, the model simulates a release of ∼2.27 ng in response to 2 seconds of stimulation at 50 spikes/s. This increase necessitated an increase in the scaling factor for the pool refill rate, *β*, from 50 to 120.

### Spiking plus secretion model

**Figure 5.**
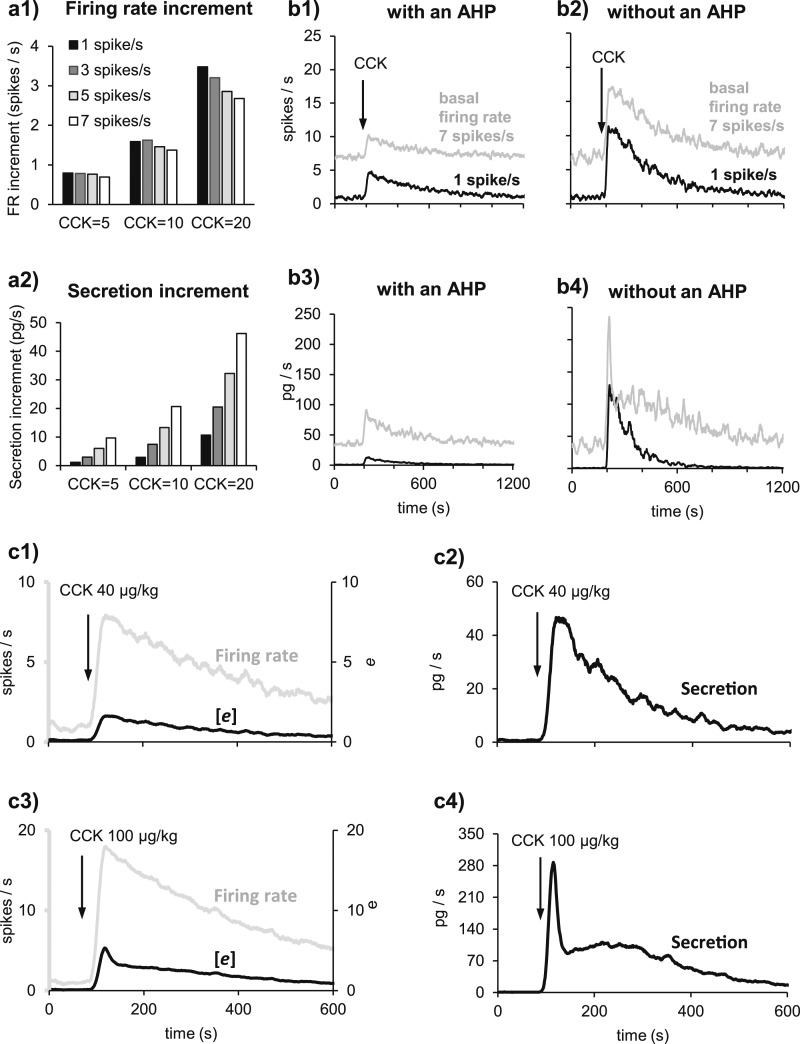
Oxytocin spiking and secretion response to CCK. (a1) Model oxytocin neurons respond to CCK by increasing their spike activity independently of the basal firing rate. The graph shows the increments in spike activity of a model cell responding to simulated challenges with different amounts of CCK (5, 10, and 20 µg/kg), from different basal firing rates (1, 3, 5, and 7 spikes/s). The same model neuron was tested 20 times, receiving random PSPs with the same average rate of 165, 348, 583, and 895 Hz. (a2) In the same simulations as in (a1), the secretion response depends on the basal firing rate. (b1) Large differences in basal firing rate (1 spike/s in black, 7 spikes/s in gray) do not drastically change the firing rate response to CCK. (b2) The same simulations as in (b1) but in a model cell without an AHP. The presence of an AHP greatly reduces the response to CCK. (b3 and b4) Secretion corresponding to the simulations in (b1) and (b2). The evoked secretion is strongly affected by the basal firing rate due to the nonlinearity of the secretion response. In (b4), the secretory response to CCK in a model cell with a basal firing rate of 7 spikes/s and no AHP shows a marked initial peak. (c1) When a model cell firing at 1 spike/s is challenged with a large CCK injection (40 µg/kg), the firing rate response closely follows the change in the model variable *e*. (c2) The secretory response to the challenge illustrated in (c1). (c3) In response to a still larger challenge (100 µg/kg CCK), the firing rate response has a similar shape as in (c1). However, *e* follows an acute initial increment that is not maintained, due to the negative feedback provoked by [Ca^2+^]. (c4) Due to those changes in *e*, amplified because secretion is proportional to *e*^2^, oxytocin secretion follows the biphasic pattern observed in (b4). FR, firing rate.

We used the combined model to explore how the spiking response to CCK varies with the dose of CCK and with the basal firing rate, and how that response affects secretion. We chose basal firing rates 1, 3, and 5 spikes/s, spanning the range in the reference data, and simulated CCK injections of 5, 10, and 20 µg/kg. Each combination was run 20 times (differing by the random differences in PSP arrival times), and the results were averaged. Each response was calculated as the difference between the average firing rate in the 25 seconds after the peak response and the basal firing rate (determined after allowing enough time for the model simulation to reach equilibrium of secretion). Comparing responses for the same CCK dose and different basal firing rates (Fig. 5a1), the response to CCK is largely independent of the basal firing rate in the range 1 to 7 spikes/s. At 20 µg/kg, where there is the biggest difference, the response from a basal firing rate of 1 spike/s (3.5 spikes/s) is 30% greater than that from a basal rate of 7 spikes/s (2.7 spikes/s).

This consistency in firing rate responses to a given dose of CCK is not present in the secretory response, similarly calculated as the difference between basal levels and evoked levels. At higher basal firing rates, the secretory response is much greater than from a basal firing rate of 1 spike/s for all doses of CCK (5, 10, and 20 µg/kg; Fig. 5a2). The relationship between EPSP rate and firing rate in the oxytocin cell model is approximately linear over the range modeled here (7) so the firing rate increment in response to CCK is relatively independent of the basal firing rate. However, the frequency dependence of stimulus-secretion coupling makes the secretory response to CCK nonlinearly dependent on the absolute firing rate achieved in response to CCK. Hence the secretory response to CCK depends on both the basal firing rate and the dose of CCK.

The influence of the AHP was examined by comparing the response of the model with and without an AHP (*i.e.*, setting *k*_AHP_ = 0) for a CCK injection of 20 µg/kg. With an AHP, the spiking response to CCK (about 4 spikes/s at peak; Fig. 5b1) is much less than without an AHP (about 11 spikes/s; Fig. 5b2). In the presence of an AHP, the profile of secretion follows that of spike activity smoothly (Fig. 5b3). By contrast, without an AHP, when the basal firing rate is 7 spikes/s, the initial high level of secretion evoked by CCK rises sharply from 40 pg/s to 235 pg/s in response to CCK, but decreases abruptly to 110 pg/s within 20 seconds before declining more smoothly (Fig. 5b4).

To understand this behavior, we simulated a similarly large spike response in the presence of an AHP. From a basal firing rate of 1 spike/s, a simulated CCK injection of 40 µg/kg evoked a response of 8 spikes/s (Fig. 5c1) and was accompanied by a smooth secretory response (Fig. 5c2). A larger CCK injection (100 µg/kg) evoked a response of 18 spikes/s (Fig. 5c3), and the accompanying secretory response rose sharply and decreased abruptly (Fig. 5c4), as observed in the model without an AHP (Fig. 5b4). This feature is because of the fatigue associated with Ca^2+^-induced inhibition of Ca^2+^ entry (Fig. 5c1 and 5c3), which becomes noticeable only above firing rates of 13 spikes/s. Thus, in the model, an abrupt pulse of oxytocin secretion can arise at the onset of a sustained increase in activity to a level exceeding 10 spikes/s.

### The diffusion model

**Figure 6.**
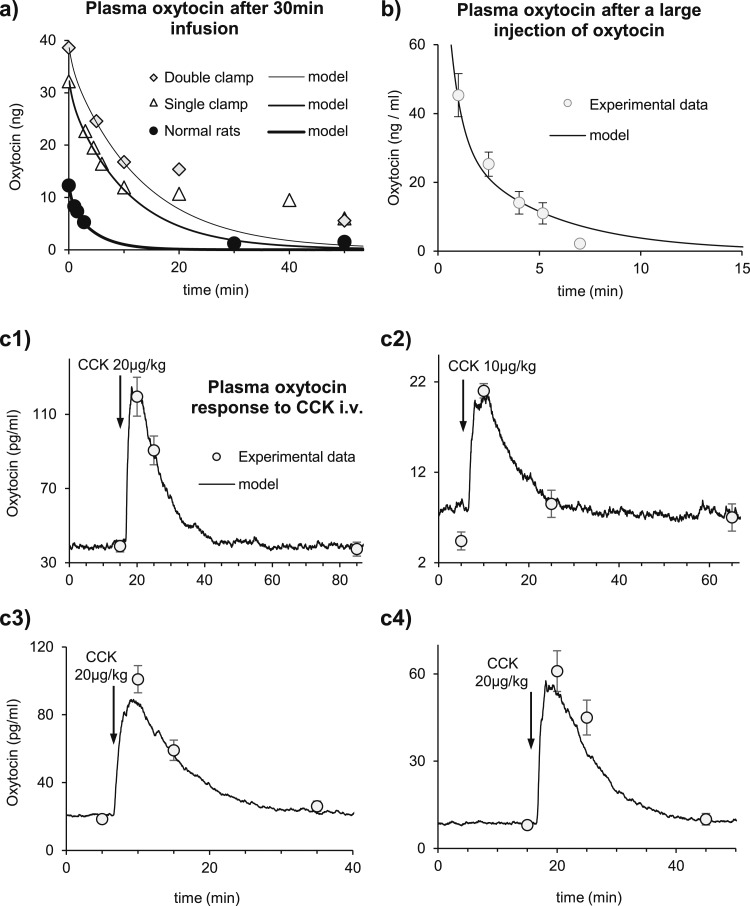
Two-compartment diffusion model and oxytocin plasma response to CCK. We calibrated our two-compartment diffusion model with data from two i.v. oxytocin protocols: after a long infusion and after a bolus injection. We then simulated the plasma oxytocin response to CCK to compare with four experimental data sets. (a) Oxytocin clearance after 30-minute infusion of 3 ng/100 g body weight/min oxytocin. Black dots, white triangles, and light-gray diamonds show the plasma oxytocin measured (29) in normal rats (black circles), rats with the kidneys or the splanchnic area clamped (gray triangles), and rats with both set of organs clamped (gray squares). Thick, normal, and thin black lines are the model results following simulations of the same protocol. The parameter values are given in Table 3. (b) Mean (standard error) plasma oxytocin after bolus (2-second) injection of 440 µg/100 g of oxytocin (28) (white dots) matched (black line) by a model with the same parameter values as used in (a). (c) Adding the diffusion model to the spiking and secretion models (solid lines), we can match the CCK experimental response in plasma (white circles with standard error shown) from four data sets by changing the basal PSP rate to match the first experimental point, and emulating the amount of CCK injected. (c1) Data from 23 conscious female rats injected with 20 µg/kg CCK (38). (c2) Data from 5 to 10 conscious male rats given 10 µg/kg CCK (19). (c3 and c4) Anesthetized female rats injected with 20 µg/kg CCK [from (39)]. Modeled data are shifted by 60 seconds in (c1) to (c4), assuming that the CCK injections were given slowly. The other model parameters are as obtained to match the spiking response to CCK (Table 1).

To model the oxytocin concentration in plasma, we simulated experiments that measured the half-life of oxytocin in plasma and its apparent volume of distribution following i.v. infusions of oxytocin in normal rats and in rats where the blood supply to the kidneys and/or the splanchnic was clamped (Fig. 6a). We created a two-compartment model where oxytocin secretion first enters a plasma compartment with volume *C_plasma_* = 8.5 mL, from which it is cleared and from which it diffuses to an extravascular compartment of volume *C_EVF_* = 9.75 mL (Fig. 1); these volumes are as estimated by Fabian *et al.* (29). We chose a diffusion half-life (*λ_diff_* = 61 seconds) compatible with the diffusion of NaCl between plasma and extravascular fluid (40). With those parameters, we matched the experimental data from continuous infusion studies (29) with a clearance half-life *λ_clr_* = 68 seconds (Table 3). Using the same parameter values, we can also match the observed oxytocin clearance after a bolus injection of oxytocin (28) (Fig. 6b).

### Spiking, secretion, and diffusion models combined

Combining the spiking, the secretion, and the diffusion models, we tried to match the oxytocin plasma concentration measured in experiments where CCK was injected in rats. In the first experiment (38), 23 rats were injected with 20 µg/kg CCK. In the second, seven rats were injected with 10 µg/kg CCK (19). Lastly, two groups of 39 and 25 rats were injected with 20 µg/kg CCK (39). In all cases, oxytocin was measured by radioimmunoassay, but in the first, third, and fourth cases, the assay used was that of Higuchi *et al.* (41), and oxytocin was measured in unextracted plasma. The second set of data measured oxytocin after plasma extraction using a different antibody (19).

We matched the first set of data (Fig. 6c1) by simulating a basal firing rate of 2.4 spikes/s, increasing the EPSP rate after 5 minutes with a simulated injection of 20 µg/kg CCK (*i.e.*, by the amount determined by the fits of the spiking model to neuronal responses to CCK; Fig. 2). In the second set of data, the oxytocin concentration did not return to the original baseline level after the CCK injection, and we simulated a basal firing rate of 0.8 spikes/s that fit the final oxytocin concentration, not the initial concentration (Fig. 6c2). In the third set of data, we simulated a basal firing rate of 1.7 spikes/s to match the average of initial and final oxytocin concentrations (Fig. 6c3), and in the final set, we matched initial and final oxytocin concentrations with a basal firing rate of 0.9 spikes/s (Fig. 6c4). For each set of data, there is a close match to the oxytocin concentrations measured in the 15 minutes after CCK injection.

### The role of the AHP

**Figure 7.**
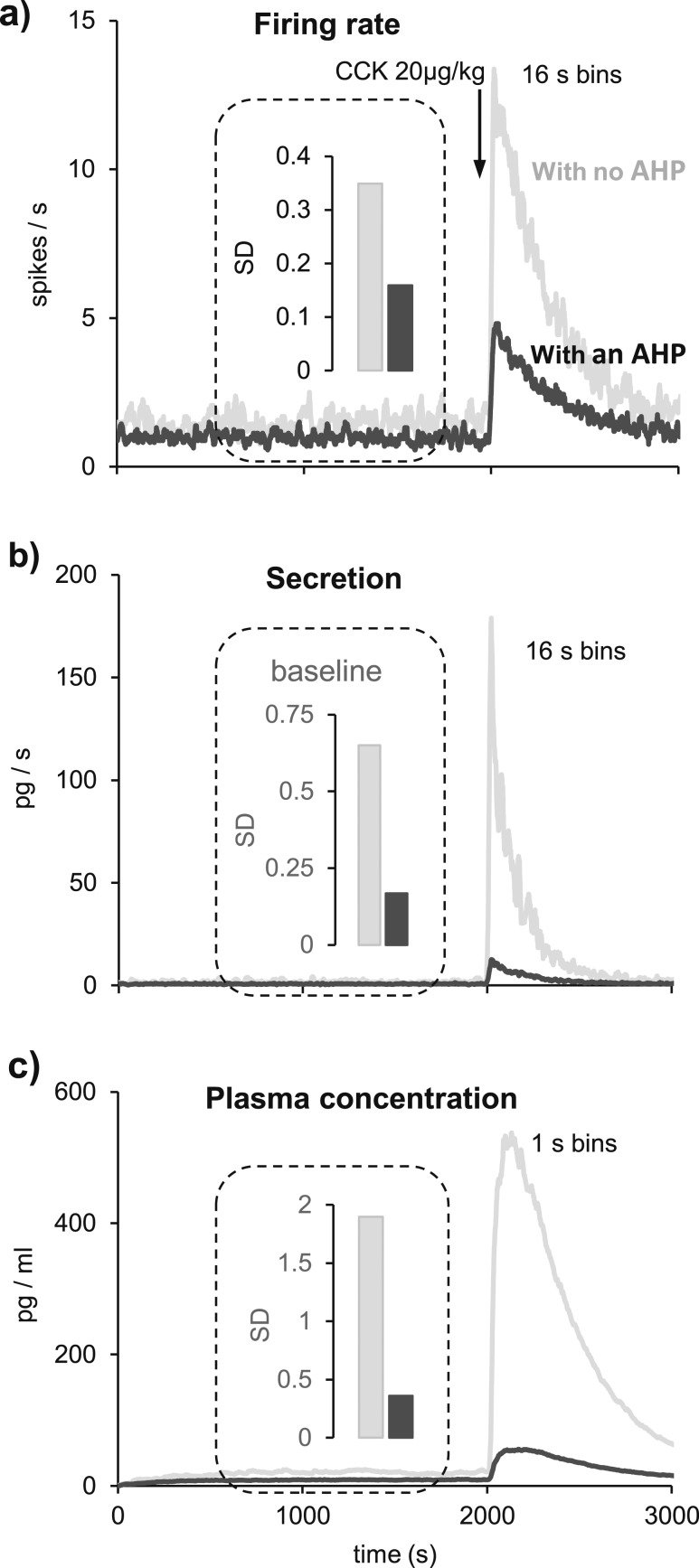
The effect of the AHP. The AHP affects the baseline behavior and the response to CCK (20 µg/kg i.v.) in the (a) spike rate, (b) secretion, and (c) plasma concentration. SD refers to the SD during the 1200-second period before CCK injection. (a) The model was set to produce a basal mean firing rate of 1 spike/s, which increased to 4.9 spikes/s after CCK (black). Removing the AHP (gray), but keeping the same initial PSP rate, produces a slightly higher basal firing rate and a much greater response to CCK. The basal firing rate, measured between 800 and 2000 seconds, is less variable with an AHP than without it as apparent from the SD (bars). (b) Secretion is a nonlinear function of the firing rate. When there is a fast change in firing rate, the nonlinearity provokes a very much larger secretory response in a model without an AHP. The basal secretion rate is much less variable with an AHP than without it. (c) Without an AHP, the oxytocin plasma concentration increases largely in response to CCK injection. Before CCK, the basal oxytocin concentration in plasma is much less variable with an AHP than without it.

As shown previously (21), the AHP “smoothes” the firing rate of oxytocin cells, reducing its variability, and it reduces the amplitude of the response to CCK (Fig. 7a). As we predicted, the AHP has an even bigger effect in reducing the variability (SD) of basal oxytocin secretion (Fig. 7b) and basal oxytocin concentrations (Fig. 7c).

**Figure 8.**
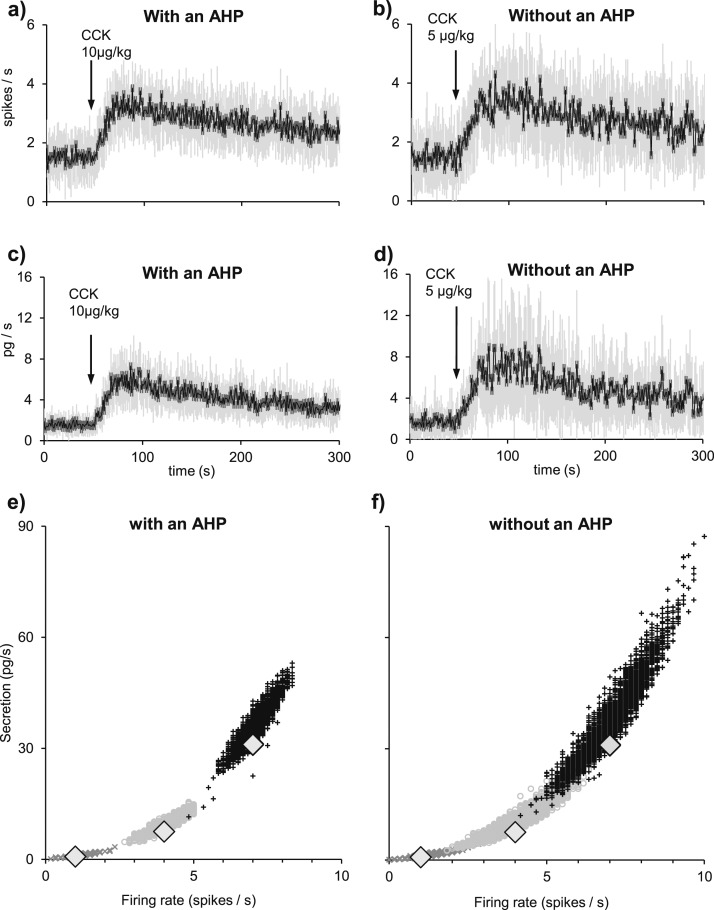
The role of the AHP in signal response. (a and b) Average firing rate of 20 runs of the same model cell. We compare average spiking and secretion responses to CCK when (a and c) there is an AHP in the model and when (b and d) there is not. Means are in black and SD in gray. (a) Modeling a basal firing rate of 1.5 spikes/s with a PSP rate of 210 Hz and an AHP. In response to a simulated injection of 10 µg/kg, the model cell responds with an increment of ∼2 spikes/s. (b) To obtain a similar average response without an AHP, we reduced the PSP rate to 165 Hz and the CCK injection to 5 µg/kg. Although the mean responses are similar, the SD (gray) is much larger without an AHP. (c and d) Secretion rates accompanying the firing rate simulations in (a) and (b). Secretion shows an even bigger difference in variability without an AHP due to the nonlinearity between secretion and firing rate. (e) Another way to see the role of the AHP is to look at oxytocin secretion in 4-second intervals in response to PSPs at a constant mean rate. In models with and without an AHP, PSP rates were chosen to produce mean firing rates of 1, 4, and 7 spikes/s (large diamonds). Because of the randomness of PSP arrival times, the firing rate varies from interval to interval around these means, and this variability is greater without an AHP. This variability is amplified by the nonlinearity of stimulus-secretion coupling. The dot clouds represent the firing rates in 6-second intervals and the concurrent secretion during 1200 seconds when the modeled neuron produces average firing rates of 1 (dark-gray crosses), 4 (gray circles), and 7 spikes/s (black pluses). In a model with an AHP, the firing rates and secretion measured in every interval both consistently distinguish the three levels of PSP rate. In a model without an AHP, there is extensive overlap.

We went on to study why this reduction in variability might be important. We used the model to mimic the same firing rate response to a simulated challenge with and without an AHP. We ran the model with an AHP 20 times (randomizing the PSP arrival times) to produce an average basal firing rate of 1.5 spikes/s and simulated the response to 10 µg/kg CCK. Then, we ran the model without an AHP, adjusting the mean PSP rate to produce the same average basal firing rate, and challenged it with a simulated injection of 5 µg/kg CCK to evoke a similar firing rate response. Although the firing rate responses to CCK are similar in magnitude, they differ in variability. With an AHP, the mean (SD) second-by-second coefficient of variation of firing rate (SD/mean) in the 20 runs for the 50 seconds before CCK plus the 300 seconds after is 0.43 (0.11) (Fig. 8a), compared with 0.60 (0.13) for the model without an AHP (Fig. 8b). For secretion, the corresponding coefficient of variation is 0.54 (0.12) in the model with an AHP (Fig. 8c), compared with 0.81 (0.17) in the model without an AHP (Fig. 8d).

To illustrate how this reduction in variability helps to distinguish between different levels of mean activity, we ran the model with and without an AHP for 20 minutes at mean firing rates of 1, 4, and 7 spikes/s. The modeled secretion varies according to the history of activity and secretion, so we plotted the actual firing rate in each 6-second bin against the secretion in that bin (Fig. 8e and 8f). In the model with an AHP, the rates of secretion are consistently separated (Fig. 8e), but in the model without an AHP, they overlap substantially (Fig. 8f).

**Figure 9.**
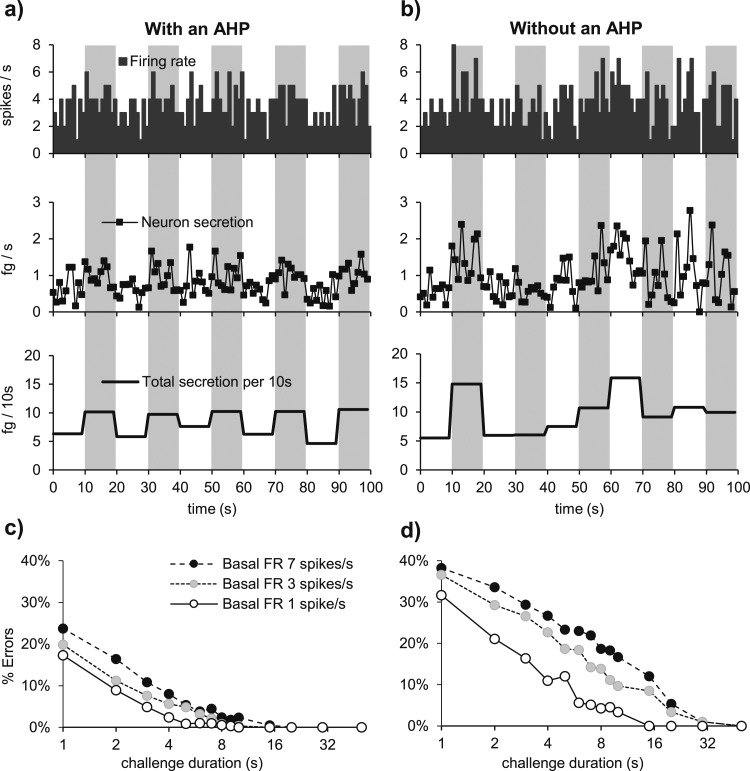
The role of the AHP in the detection of a transient signal. (a) We ran the model as a single neuron with a mean basal firing rate (FR) of 1, 3, and 7 spikes/s. By modifying the EPSP rate, we raised the firing rate by an average of 1 spike/s for a 10-second challenge episode every 20 seconds for 100 minutes. The graphs plot the model outputs (firing rate, secretion per second, and secretion per 10 seconds) for a model neuron firing at 3 spikes/s for the first 100 seconds of the simulation. (b) As in (a), but for a model with no AHP. Note that in (a), episodes of greater EPSP rate are consistently associated with greater secretion (as measured in 10-second bins), but this is not true of a model with no AHP. (b–e) Similar experiments to those in (a) and (b) were performed to assess challenge episodes of different duration (1, 3, 5, 10, 15, 30, and 50 seconds). (c) Shows the percentage of errors for a neuron firing at a basal firing rate of 1, 3, and 7 spikes/s when there is an AHP. (d) Same but without an AHP. In all cases, there is a much smaller error rate when there is an AHP.

Finally, we considered how the AHP affects the reliability of the signal from a single oxytocin neuron. We ran the model with and without an AHP, as a single neuron firing on average at 1, 3, and 7 spikes/s. We calculated what increase in EPSP rate in each condition would raise the mean firing rate by 1 spike/s on average. We then tested the model with a pattern of EPSPs that alternated between the higher (challenge) and lower (basal) level for different durations (1 to 50 seconds) and for a total run of 100 minutes (Fig. 9) and compared the modeled total secretion during each challenge episode with that in the preceding basal episode. If the secretion during the challenge episode did not exceed that during the corresponding basal episode, we registered this as a “detection error” and counted the number of such errors in each trial.

The error rate is consistently much higher without an AHP: for example, at 3 spikes/s, 1-second challenge episodes are not detected in 37% of trials of a neuron without an AHP, but in only 20% of trials of a neuron with an AHP (Fig. 9c and 9d).

### Heterogeneity in basal firing rate and response to CCK

**Figure 10.**
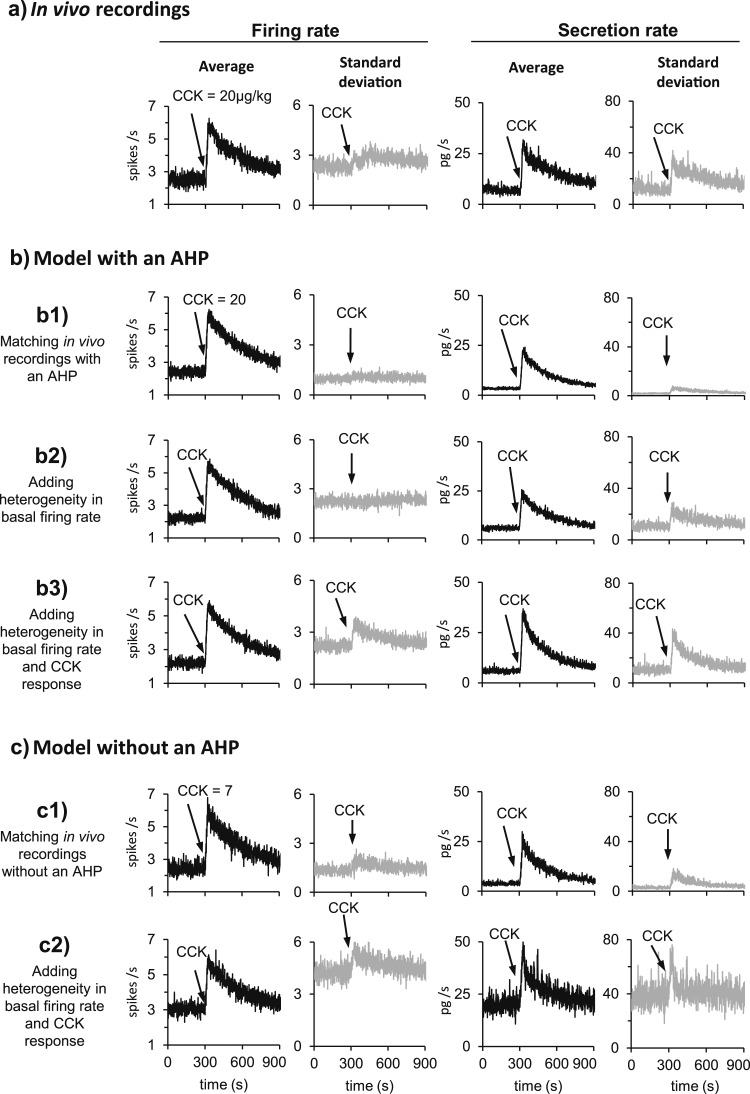
Heterogeneity in oxytocin spiking activity and response to CCK. From left to right, each set of panels shows the average firing rate of the 23 neurons (in 1-second bins), the SD of the firing rate, the predicted *in vivo* secretion (in 1-second bins), and the SD of the predicted secretion. (a) Data from the 23 reference neurons. (b1) Averages of 23 runs of the model with random PSP arrival times at a fixed PSP rate. The SD of the basal firing rate is much smaller than in (a), and does not increase after CCK. The basal secretion is lower than in (a), but the increment after CCK (∼20 pg/s) is similar. The SD in the model is also much lower than in (a). (b2) To simulate heterogeneity in oxytocin neurons, we varied the basal PSP rate using a log-normal distribution. The heterogeneity elevates the SD to the level in (a), but the SD still does not increase after CCK. The secretion is close to that in (a). (b3) Adding heterogeneity to the response to CCK gives a very close match to all panels in (a). (c1) In 23 homogeneous model cells without an AHP, a match to the mean firing rates of the reference neurons is obtained by reducing the basal PSP rate and *k*_CCK_. The SD of the firing rate is lower than in a but higher than in (b1). The basal secretion and the SD of secretion are lower than in (a). (c2) Adding heterogeneity to the model without an AHP produced a higher basal firing rate and smaller response to CCK, but greatly increased the SD of the firing rate and the secretion rate to levels much higher than in (a). Thus a close match to both the mean and the variability of the (a) reference data was obtained by modeling a population of neurons matching the heterogeneity of those data using a model with an AHP, but not by using a model without an AHP. The parameter changes used for the simulations shown are given in Table 5.

In the reference data, the basal firing rate and the response to CCK are both heterogeneous: The mean SD of the basal firing rate (in 1-second bins) was 2.34, close to the SD = 2 reported previously for oxytocin neuron firing rates (37). This increased to 2.84 over the 5 minutes after injection of CCK, also consistent with previous data (15). To evaluate how that heterogeneity affects secretion, we ran the model with the recorded spike times of those 23 neurons, obtaining the predicted secretion and its mean SD (Fig. 10a).

In Fig. 2, we simulated the average response of those 23 neurons using 23 runs of a single model neuron with randomized input arrival times at the same mean rate. This gives a much lower SD of firing rate (Fig. 10b1). Moreover, although the averaged modeled secretion was close to the predicted *in vivo* secretion (Fig. 10b1), the mean basal secretion (3.34) is much lower than the predicted mean basal secretion (7.11). That leads to an overestimate of the predicted basal firing rate when fitting the model to plasma oxytocin measurements, as we did in Fig. 6c. The SD of the secretion rate was also much lower.

Therefore, we introduced heterogeneity in our model by simulating a population of 23 neurons with independently generated values for PSP rate [*I_re_* and *I_ri_* sampled from a log-normal distribution with mean (SD) = 292 (292); see Table 5] to closely match both the average firing rate of the reference neurons and the SD of their basal firing rate (Fig. 10b2). However, the SD of the firing rate of the heterogeneous model cells did not change after CCK (2.23 at basal level and 2.24 after CCK). Consequently, the secretion rate is similar to the predicted secretion from the reference neurons at the baseline, but does not reach the predicted levels of CCK response.

**Table 5.** Spiking Parameter Values to Simulate Heterogeneity in an Oxytocin Neuron Population (Fig. 10)

Neuron	With an AHP						Without an AHP			
	Fixed Basal Input Rate and CCK Response		Random Basal Firing Rate		Random Basal Firing Rate and CCK Response		Fixed Basal Firing Rate and CCK Response		Random Basal Firing Rate and CCK Response	
	*I_re_* and *I_ri_*	*k_CCK_*	*I_re_* and *I_ri_*	*k_CCK_*	*I_re_* and *I_ri_*	*k_CCK_*	*I_re_* and *I_ri_*	*k_CCK_*	*I_re_* and *I_ri_*	*k_CCK_*
1	292	20	112	20	112	13.9	203.5	7	71	16.1
2	”	”	63	”	63	7.5	”	”	322	3.7
3	”	”	68	”	68	5.6	”	”	101	2.5
4	”	”	92	”	92	55.3	”	”	490	1.7
5	”	”	180	”	180	18.0	”	”	71	5.2
6	”	”	74	”	74	12.0	”	”	292	6.1
7	”	”	278	”	278	8.2	”	”	208	5.1
8	”	”	488	”	488	5.5	”	”	85	8.0
9	”	”	203	”	203	8.0	”	”	43	6.5
10	”	”	69	”	69	8.7	”	”	335	5.4
11	”	”	237	”	237	10.5	”	”	587	12.3
12	”	”	279	”	279	16.8	”	”	104	3.8
13	”	”	411	”	411	41.0	”	”	622	1.1
14	”	”	156	”	156	14.0	”	”	114	13.2
15	”	”	494	”	494	23.4	”	”	83	25.5
16	”	”	383	”	383	41.7	”	”	63	3.9
17	”	”	218	”	218	35.1	”	”	240	4.0
18	”	”	699	”	699	2.3	”	”	36	3.5
19	”	”	204	”	204	27.3	”	”	410	12.7
20	”	”	1026	”	1026	20.6	”	”	104	3.2
21	”	”	67	”	67	15.8	”	”	127	5.0
22	”	”	335	”	335	38.9	”	”	129	10.8
23	”	”	574	”	574	37.2	”	”	45	2.0
Average	292	20	291.7	20	291.7	20.3	203.5	7	203.7	7.0

We therefore also introduced heterogeneity to the CCK response by simulating a log-normally distributed *k_CCK_* of mean (SD) = 20 (20). With that change, the match to the firing rate was still good, and the SD of the basal firing rate (2.21) and the SD during the 5 minutes after CCK (2.93) were close to the reference data (Fig. 10b3). In addition, the model mimicked the predicted *in vivo* secretion closely in both average and SD (see Table 5 for the parameter values of the 23 neurons).

To study the impact of the AHP, we made the AHP = 0, and in the model without heterogeneity, we reduced the basal PSP rate from 292 to 203.5 and *k_CCK_* from 20 to 7 to match the mean firing rate of the reference data (Fig. 10c1). The resulting SD of the firing rate was 1.33 at the basal level and 1.71 after CCK (Fig. 10c1). The secretion still matched the predicted *in vivo* reference data but not the SD. We then introduced heterogeneity by varying the PSP rate [log-normal distribution with mean (SD) = 203.5 (203.5)] and *k_CCK_* [log-normal distribution with mean (SD) = 7 (7)]. This gave a raised mean basal firing rate of 3.06 spikes/s and a mean response magnitude of 1.2 spikes/s over the 5 minutes after CCK (Fig. 10c2). The firing rate SD was 4.27 at basal and 4.9 after CCK, much higher than in the reference data. The close match to predicted secretion was lost (see Table 5 for the parameter values of the 23 neurons without an AHP). Thus a close match to both the mean and the variability of the reference data (Fig 10a) was obtained by modeling a population of neurons matching the heterogeneity of those data using a model with an AHP, but not by using a model without an AHP.

## Discussion

In the current study, we used a previously published integrate and fire–based model of oxytocin neuron activity. We have shown elsewhere that this model is closely consistent with a Hodgkin-Huxley–type model that represents the AHP and HAP in a biophysically meaningful way, consistent with experimental data from *in vitro* experiments (7). Real oxytocin neurons vary in their intrinsic properties, in their basal firing rates, and in their responsiveness to CCK. In the current study, we began by considering the population of oxytocin neurons as identical, differing in their behavior only as a result of differences in the random arrival times of PSPs. The model neurons are however “typical” of real oxytocin neurons, and we simulated a response to CCK that matches the average response of real neurons to CCK. We did so by the minimalist assumption that the mean rate at which EPSPs arrive is proportional to the CCK concentration in plasma, which decays exponentially to zero after bolus injection. The decay of plasma CCK estimated in the current study from the recordings of oxytocin cells (230 seconds) is close to the half-life of CCK measured in human plasma (about 4 minutes) (42).

To this spiking model, we added a model of stimulus-secretion coupling adapted from a model used previously to model stimulus-secretion coupling in vasopressin neurons. We adapted that model to match four sets of published data on oxytocin secretion from isolated posterior pituitaries.

The model expresses the rate of oxytocin secretion from a single neuron as a continuous variable. This understates the variability of secretion from a single cell. Oxytocin is secreted in discrete packets (vesicles that each contain about 85,000 molecules of oxytocin), and the rate at which these vesicles are secreted from a single cell at baseline is low (about 1 to 4/s) (43). A more accurate model would represent secretion as a stochastic process, not as a continuous deterministic process. In the context of the present model, we can better understand the variable (*s*), described here as representing the rate of secretion, as rather reflecting the instantaneous probability of vesicle exocytosis. However, as we are using this model to simulate the total secretion from the population, it seemed reasonable to accept a continuous representation of secretion as approximating the average of many stochastic processes.

The secretion model is a highly simplified representation of mechanisms in the nerve terminals. The terminals express a variety of Ca^2+^ channels (44), and there is evidence that Ca^2+^ release from intracellular stores also has a role (45). Exocytosis is also modulated by activity-dependent secretion of several modulators, including adenosine triphosphate (46), adenosine (47), and endogenous opioids (48, 49). The terminals do not contain clearly separate pools of readily releasable and reserve vesicles, but rather a heterogeneous population differing in releasability (50). Other mechanisms also affect stimulus-secretion coupling, including changes in axonal excitability that result from activity-dependent changes in extracellular potassium concentration (51, 52). In this study, our purpose was not to construct a detailed model of all of the mechanisms that contribute to stimulus-secretion coupling, but rather to produce a minimalist model that by matching available data on stimulus-secretion coupling would enable us to predict secretion from spiking activity (31, 52–54).

Combining the spiking model with this secretion model allowed us to model the activity-dependent output of oxytocin cells. To relate this to measurements of secretion *in vivo*, we needed to scale the output of the model by choosing an appropriate value for the scaling factor *α*. The *in vitro* measurements used to fit the model report variable absolute levels of oxytocin secretion; in these experiments, glands are impaled on stimulating electrodes, and exactly where the glands are impaled will determine what proportion of the axons are stimulated, and there will be variable damage to axons. Stripping neural lobes from the adjacent intermediate lobe reduces the volume of tissue impaled and makes it more likely that the tissue is effectively stimulated, but entails greater tissue damage. In such experiments (55), 6 minutes of stimulation at 13 Hz released up to 9 ng oxytocin. Matching this with the secretion model suggests a value of *α* = 0.0015 as a lower bound of plausible values. Wakerley and Lincoln (22) estimated stimulus-evoked release in lactating rats by stimulating the neural stalk and comparing the resultant increase in intramammary pressure with that evoked by i.v. injections of oxytocin: They estimated that about 1 mU was released by 4 seconds of stimulation at 50 Hz, consistent with *α* = 0.002. However, the response to stimulation *in vivo* varies with the precise placement of the electrode and with the stimulus current used. There is one circumstance, which does not involve that uncertainty. In the anesthetized lactating rat, suckling evokes intermittent milk ejection bursts in oxytocin neurons, which typically contain between 50 and 100 spikes and which last for about 2 seconds. These bursts produce an abrupt rise in intramammary pressure that can be mimicked by i.v. injection of 0.5 to 1 mU oxytocin (1.1 to 2.2 ng). The release of 2.2 ng of oxytocin by 2 seconds of stimulation at 50 Hz in the model is closely approximated by a value of *α* = 0.003, which was chosen for subsequent tests of the model.

To predict the consequences of oxytocin secretion for plasma concentrations, we also needed to model the clearance of oxytocin. Early studies in the rat studied clearance in two ways: by infusing oxytocin continuously and measuring the achieved concentration at equilibrium and the decline after stopping infusion (29), and by injecting large amounts as a bolus and measuring the decline (28). In the former case, experiments studied the mechanisms of clearance by clamping vessels to the kidneys and splanchnic area. These two sets of data could mostly be well matched by a two-compartment model, except that, in data from rats with venous clamps, oxytocin concentrations remained elevated above predicted levels in a way that Fabian *et al.* (29) proposed that arose from a time- and surgery-dependent increase in the apparent distribution volume. This, we did not attempt to mimic in the model.

Having selected a value for *α*, and with a validated model for plasma clearance of oxytocin, we could use the model to predict the changes in plasma concentration that result from the response of oxytocin neurons to CCK. For this, we had four sets of data in which oxytocin was measured in different conditions (conscious and anesthetized rats; male and female rats) at two different doses of CCK and using two different radioimmunoassays.

Matching the initial basal level of secretion in these data implied differences in the basal firing rates of oxytocin neurons in the four conditions. The mean basal firing rates inferred from the model were 2.4 spikes/s for conscious female rats (Fig. 6c1), 0.8 spikes/s for conscious male rats (Fig. 6c2), and 1.7 spikes/s and 0.9 spikes/s for urethane-anesthetized female rats (Fig. 6c3 and 6c4). These are lower than the basal firing rates recorded from the SON of urethane-anesthetized virgin female rats, which are generally about 2.5 spikes/s (37, 56), but this difference is as expected, given that the electrophysiological recordings are from rats in which the hypothalamus has been exposed by transpharyngeal surgery. The trauma and blood loss entailed in this surgery increases the basal activity of both oxytocin and vasopressin neurons. Across these four sets of data, there is good agreement between model predictions of the response to CCK and experimentally measured levels.

We went on to use the model to investigate the role of the AHP. This activity-dependent potential, which is pronounced after high frequency spiking, is important in shaping the profile of milk ejection bursts (57). However, the AHP is also active at low basal firing rates, and it both restrains basal activity and reduces the variability of firing rate (21). Secretion is coupled nonlinearly to firing rate, and as a result, variability of firing rate produces an amplified variability of secretion.

The extent of this variability is illustrated in Fig. 8, which shows that for an oxytocin neuron firing at 7 spikes/s, the secretion in 6-second bins is very variable, but is always distinct from the secretion resulting from a firing rate of 4 spikes/s. However, for a neuron without an AHP, there is considerable overlap: The mean spike rate cannot be reliably estimated from the secretion measured in a given 6-second bin. This variability is of little consequence to plasma levels in the rat: Oxytocin in plasma has a half-life of about 1 minute, and levels reflect the activity of about 9000 neurons. However, for smaller animals, such variability may be more problematic. In zebrafish for example, the ortholog of oxytocin, isotocin, is expressed in only a few tens of neurons (58).

In mammals, small subsets of magnocellular neurons project to diverse sites in the brain, and at these sites, stability of oxytocin secretion rates might also be important. We have shown (Fig. 9) that even changes in spike activity that have large functional consequences (1 spike/s) cannot be consistently detected as changes in secretion from a single neuron unless secretion is averaged over many seconds. Without an AHP, only the average secretion over 30 seconds will consistently reveal a rise in mean firing rate. Thus, the output of a single oxytocin neuron is a very noisy reflection of the signal that determines the mean level of its afferent inputs, but the presence of an AHP markedly enhances its signal detection ability. This may be important for a small population of neurons, including for the small subsets of oxytocin neurons that project to various forebrain sites, but is much less important for the large population that projects to the pituitary.

An important consideration in inferring physiological significance to the behavior of a single neuron is how population heterogeneity may temper those inferences. Oxytocin neurons are certainly heterogeneous—in their basal firing rates, in their responsiveness to physiological stimuli, and in their intrinsic membrane properties, including those that determine the HAP and the AHP.

Here we simulated some of this heterogeneity by running our single neuron model with varied synaptic input rates, and by introducing variability into the simulated CCK challenge. The activity dependence of the AHP means that it has a stronger inhibitory effect on more active neurons, pulling them closer to the mean firing rate. Thus, as well as reducing signal variability within single neurons, the AHP reduces the variability of firing rate of a heterogeneous population (Fig. 10), with an even larger effect on the variability of secretion rate because of the nonlinear coupling of firing rate to secretion.

A full appreciation of the effects of heterogeneity is beyond the scope of the present paper. It remains to be determined how variability in intrinsic properties interacts with variability in input rates, and how variability in the population signal affects secretion might depend on assumptions about the independence of input signals. Each oxytocin neuron receives many synaptic inputs, and it is likely that these are from overlapping subsets of a larger presynaptic population, resulting in many neurons receiving the same input noise. There is extensive data already in the literature on both the electrophysiological responses of oxytocin neurons to different stimuli and on associated plasma oxytocin responses, giving a potentially rich source of material to test and refine the present model.

## Abbreviations:

AHP: afterhyperpolarization
CCK: cholecystokinin
EPSP: excitatory postsynaptic potential
HAP: hyperpolarizing afterpotential
IPSP: inhibitory postsynaptic potential
i.v.: intravenous
PSP: postsynaptic potential
SD: standard deviation
SON: supraoptic nucleus
